# Mitochondrial genome characterization of the family Trigonidiidae (Orthoptera) reveals novel structural features and *nad1* transcript ends

**DOI:** 10.1038/s41598-019-55740-4

**Published:** 2019-12-13

**Authors:** Chuan Ma, Yeying Wang, Licui Zhang, Jianke Li

**Affiliations:** 1 Institute of Apicultural Research/Key Laboratory of Pollinating Insect Biology, Ministry of Agriculture, Chinese Academy of Agricultural Sciences, Beijing, 100093 China; 2 Key Laboratory of State Forestry Administration on Biodiversity Conservation in Karst Mountainous Areas of Southwestern China, Guizhou Normal University, Guiyang, 550025 China

**Keywords:** Comparative genomics, Mitochondrial genome

## Abstract

The Trigonidiidae, a family of crickets, comprises 981 valid species with only one mitochondrial genome (mitogenome) sequenced to date. To explore mitogenome features of Trigonidiidae, six mitogenomes from its two subfamilies (Nemobiinae and Trigonidiinae) were determined. Two types of gene rearrangements involving a *trnN*-*trnS1*-*trnE* inversion and a *trnV* shuffling were shared by Trigonidiidae. A long intergenic spacer was observed between *trnQ* and *trnM* in Trigonidiinae (210−369 bp) and Nemobiinae (80–216 bp), which was capable of forming extensive stem-loop secondary structures in Trigonidiinae but not in Nemobiinae. The anticodon of *trnS1* was TCT in Trigonidiinae, rather than GCT in Nemobiinae and other related subfamilies. There was no overlap between *nad4* and *nad4l* in *Dianemobius*, as opposed to a conserved 7-bp overlap commonly found in insects. Furthermore, combined comparative analysis and transcript verification revealed that *nad1* transcripts ended with a U, corresponding to the T immediately preceding a conserved motif GAGAC in the superfamily Grylloidea, plus poly-A tails. The resultant UAA served as a stop codon for species lacking full stop codons upstream of the motif. Our findings gain novel understanding of mitogenome structural diversity and provide insight into accurate mitogenome annotation.

## Introduction

The typical mitochondrial genome (mitogenome) of insects is a circular molecule ranging in size from 15 kb to 18 kb^1^. It harbors 37 genes including two ribosomal RNA (rRNA) genes, 22 transfer RNA (tRNA) genes, and 13 protein-coding genes (PCGs). These genes are mainly arranged in a conserved order that is regarded as the ancestral gene arrangement of insects. The mitogenome also contains non-coding regulatory regions, including a control region with initiation sites for replication and transcription^1^ and, in most insect orders, an intergenic spacer (IGS) between *nad1* and *trnS2* with a conserved binding site for the transcription termination factor^2,3^. During transcription, insect mitogenome is first transcribed into polycistronic primary transcripts, which are processed into mature transcripts including 33 single gene transcripts and two bicistronic transcripts encoding the *atp8*/*atp6* and *nad4l*/*nad4* gene pairs^4^. Typically, a 7-bp overlap exists between the two genes forming the bicistronic transcripts^3^. Insect mitogenome provides an abundant source of sequence data for population and phylogenetic studies^1,3,5–8^. In addition, structural features such as gene rearrangements and stem-loop secondary structures are used as supplementary markers to trace evolutionary history^8,9^.

The Trigonidiidae is a worldwide distributed family of crickets. It encompasses two subfamilies, Nemobiinae (ground crickets) and Trigonidiinae (sword-tailed crickets), and 981 extant species^10^. Many members are well-known for calling songs and are kept as pets for entertainment in Asian and European countries, especially in China and Japan^11,12^. Despite the high species diversity of the family Trigonidiidae, its mitogenome information is severely underrepresented. To date, only the complete mitogenome of *Trigonidium sjostedti* (Chopard, 1925)^6^ from Trigonidiinae has been sequenced within the whole family. It shows unique structural features, including particularly the occurrence of two types of gene rearrangements: an inversion of *trnN*-*trnS1*-*trnE* encoded on the majority strand to *trnE*-*trnS1*-*trnN* on the minority strand and a shuffling of *trnV* to the position between *rrnS* and the control region. The inversion is also present in two closely related families, Gryllidae and Phalangopsidae, and it is thus proposed that the inversion is a synapomorphy of the three families^13^. To consolidate this proposal, an expanded mitogenome sampling is critical especially for Trigonidiidae with the single representative mitogenome available so far. As for the *trnV* shuffling, it is not reported in closely related families except for *Ornebius bimaculatus* (Shiraki, 1930)^13^. Still, it remains yet to be explored whether the *trnV* shuffling in Trigonidiidae is specific to *T*. *sjostedti* or conserved at a higher taxonomic level. Worse still, there has not been even an attempt to investigate mitogenome features of the other subfamily Nemobiinae. There is thus an urgent need of mitogenome sequencing of Trigonidiidae, which can shed light on the phylogenetic distribution and evolutionary origin of these rearrangements and other features.

To fill this gap, three representative mitogenomes (*Dianemobius fascipes* (Walker, 1869), *D*. *furumagiensis* (Ohmachi & Furukawa, 1929), and *Polionemobius taprobanensis* (Walker, 1869)) from Nemobiinae and three (*Homoeoxipha nigripes* Xia & Liu, 1992, *Natula pravdini* (Gorochov, 1985), and *Svistella anhuiensis* He, Li & Liu, 2009) from Trigonidiinae were sequenced and annotated in this study. A detailed comparison of structural features among these mitogenomes was performed and, to place the analysis in a broader context, additional mitogenomes currently available for Grylloidea (a superfamily of crickets) was included. Comparative analysis reveals gene rearrangement patterns and a series of other shared and unique structural features, contributing to our understanding of structural diversity of insect mitogenomes. More importantly, we provide a means to ensure accurate annotation of *nad1* stop codons for Grylloidea.

## Methods

### High-throughput sequencing

*D*. *fascipes* and *P*. *taprobanensis* samples were collected in field from Shandong, China, while *D*. *furumagiensis*, *S*. *anhuiensis*, *H*. *nigripes*, and *N*. *pravdini* were purchased from a flower and bird market in Shanghai, China. To determine the 3′ end of *nad1* transcript, an additional species *Truljalia hibinonis* (Matsumura, 1917) from the family Gryllidae was collected from Shanghai, China. These samples were preserved in 100% ethanol at 4 °C prior to mitogenome sequencing and were preserved directly at −80 °C before RNA analysis. Genomic DNA was extracted from a single specimen for each species using a DNeasy Blood & Tissue kit (Qiagen, Hilden, Germany). The DNA was utilized for 150-bp paired-end sequencing on the Illumina HiSeq. 2500 platform (Novogene, Beijing, China) following the manufacturer′s protocol. After removal of poor-quality reads with Trimmomatic^14^, the remaining reads were used to assemble the full mitogenome by SOAPdenovo-Trans^15^ and MITObim v1.9^16^ with parameter setting as per Ma and Li^13^.

### PCR amplification and Sanger sequencing

To avoid short-read misassembly of non-coding regions mainly due to potential presence of long tandem repeats^13^, PCR amplification (see Table S1 for PCR primers) followed by Sanger sequencing was performed spanning the control region and the IGS between *trnQ* and *trnM*. The amplification was performed with LA Taq™ (TaKaRa, Dalian, China) under the following conditions: 95 °C for 1 min; 30 cycles of 98 °C for 10 s, 50 °C for 10 s, and 65 °C for 3 min; 65 °C for 5 min. Purified PCR products were sequenced using 3730xl DNA Analyser (Applied Biosystems, CA, USA).

### Mitogenome annotation

Mitogenome annotation was performed on the MITOS webserver (http://mitos.bioinf.uni-leipzig.de/index.py)^17^ followed by manual correction. To validate *nad1* stop codons, the 3′ end of *nad1* transcripts in *D*. *furumagiensis*, *N*. *pravdini*, *P*. *taprobanensis*, *S*. *anhuiensis*, and *T*. *hibinonis* was amplified with a SMARTer RACE 5′/3′ Kit (Clontech, CA, USA) and sequenced with Sanger sequencing. Specifically, total RNA was extracted from −80 °C stored samples with TRIzol (Invitrogen, CA, USA) and used for first-strand cDNA synthesis. Rapid amplification of cDNA ends (RACE) was performed with gene-specific primers (Table S1) and the following cycling conditions: 94 °C for 30 s; 5 cycles of 94 °C for 30 s, 68 °C for 30 s, and 72 °C for 60 s; 27 cycles of 94 °C for 30 s, 65 °C for 30 s, and 72 °C for 60 s. Purified PCR products were sequenced directly using 3730xl DNA Analyser (Applied Biosystems, CA, USA). To test whether *nad4* and *nad4l* were expressed in a bicistronic mRNA, 5′ RACE of *nad4* transcript and 3′ RACE of *nad4l* transcript of *D*. *furumagiensis* were performed employing the above methods (primers are listed in Table S1). The mitogenome sequences were deposited in GenBank (Table 1).

**Table 1:** Currently available mitogenomes for the superfamily Grylloidea.

Family	Subfamily	Species	GenBank	Size	(A + T)%	AT skew	GC skew
Gryllidae	Eneopterinae	*Cardiodactylus muiri*	NC_037914	16328	76.48	0.083	−0.289
Gryllidae	Eneopterinae	*Xenogryllus marmoratus*	NC_041236	15762	72.12	0.132	−0.376
Gryllidae	Gryllinae	*Loxoblemmus doenitzi*	NC_033985	15396	73.25	0.116	−0.296
Gryllidae	Gryllinae	*Loxoblemmus equestris*	NC_030763	16314	71.91	0.122	−0.272
Gryllidae	Gryllinae	*Teleogryllus emma*	KU562917	15697	73.22	0.099	−0.283
Gryllidae	Gryllinae	*Teleogryllus commodus*	JQ686193	15870	73.69	0.081	−0.276
Gryllidae	Gryllinae	*Velarifictorus hemelytrus*	NC_030762	16123	72.65	0.093	−0.348
Gryllidae	Oecanthinae	*Oecanthus rufescens*	KX057720	15617	77.19	0.025	−0.244
Gryllidae	Oecanthinae	*Oecanthus sinensis*	NC_034799	16142	77.39	0.021	−0.249
Gryllidae	Podoscirtinae	*Truljalia hibinonis*	NC_034797	15120	75.39	0.068	−0.294
Mogoplistidae	Mogoplistinae	*Ornebius bimaculatus*	NC_039666	16136	76.47	0.024	−0.315
Mogoplistidae	Mogoplistinae	*Ornebius fuscicercis*	NC_039739	16368	74.95	0.011	−0.320
Mogoplistidae	Mogoplistinae	*Ornebius kanetataki*	NC_039667	16589	74.24	−0.004	−0.338
Phalangopsidae	Cachoplistinae	*Cacoplistes rogenhoferi*	NC_039664	16018	73.31	0.068	−0.332
Phalangopsidae	Cachoplistinae	*Meloimorpha japonica*	NC_039665	15880	72.43	0.073	−0.292
Trigonidiidae	Trigonidiinae	*Homoeoxipha nigripes*	MK303553	15679	77.63	0.028	−0.267
Trigonidiidae	Trigonidiinae	*Natula pravdini*	MG701239	15817	78.77	0.010	−0.268
Trigonidiidae	Trigonidiinae	*Svistella anhuiensis*	MG701238	16494	73.75	0.060	−0.351
Trigonidiidae	Trigonidiinae	*Trigonidium sjostedti*	NC_032077	15763	76.89	0.020	−0.287
Trigonidiidae	Nemobiinae	*Dianemobius fascipes*	MK303550	15363	72.28	−0.010	−0.338
Trigonidiidae	Nemobiinae	*Dianemobius furumagiensis*	MK303551	15350	73.77	−0.014	−0.330
Trigonidiidae	Nemobiinae	*Polionemobius taprobanensis*	MK303552	>16641	70.34	−0.015	−0.384

### Sequence analysis

Tandem repeats were identified using the online Tandem Repeats Finder with default setting^18^. Secondary structures of IGSs and *trnS1* were predicted with the Mfold^19^ and the MITOS webserver^17^, respectively, and drawn with VARNA^20^. Nucleotide composition was calculated with DAMBE^21^. AT skew [(A − T)/(A + T)] and GC skew [(G − C)/(G + C)]^22^ were used to measure strand asymmetry in nucleotide compositions. To conduct a comparative analysis at a higher taxonomic level, 16 more mitogenomes in the superfamily Grylloidea were downloaded from GenBank and combined with the six newly sequenced mitogenomes (Table 1). DNA sequences were aligned using MUSCLE^23^ with manual refinement for *nad1* and neighboring regions to arrange the incomplete stop codon T and the immediately adjacent motif GAGAC (see Results and Discussion) in a successive manner.

### Phylogenetic analysis

Four additional mitogenomes (*Gryllotalpa orientalis* Burmeister, 1838, NC_006678; *Myrmecophilus manni* Schimmer, 1911, NC_011301; *Troglophilus neglectus* Krauss, 1879, NC_011306; *Comicus campestris* Irish, 1986, NC_028062) from closely related superfamilies (Gryllotalpoidea, Rhaphidophoroidea, and Schizodactyloidea) were selected to root the tree of the superfamily Grylloidea. The 37 genes were each aligned in MUSCLE^23^ and trimmed in Gblocks^24^ before concatenation into a single dataset. Mutational saturation test in DAMBE^21^ by plotting observed substitutions against GTR-corrected genetic distances indicated severe saturation of third codon positions of the PCGs, which was also observed in a previous study^13^. The third codons were thus removed from phylogenetic analysis. Partition schemes and substitution models for each gene and codon position were selected in PartitionFinder v2.1.1^25^. Four partitions each with GTR + I + G substitution model was set for the Bayesian tree reconstruction using MrBayes v3.2^26^. The analysis was run for 10 million generations with a sampling frequency of 1000 generations and a burnin of 25%. A maximum likelihood tree was inferred using RAxML v8.2.10^27^ with the GTRGAMMA substitution model and four partitions identified in PartitionFinder v2.1.1^25^. Branch support was assessed by 1000 bootstrap replicates.

## Results and Discussion

### Mitogenome organization

Mitogenomes of six representative species belonging to the family Trigonidiidae were determined in this study. Of them, five full-length mitogenome sequences were obtained, ranging in size from 15,350 bp in *D*. *furumagiensis* to 16,494 bp in *S*. *anhuiensis* (Table 1). These sizes fell within those of typical insect mitogenomes^1,28^. *P*. *taprobanensis* had a larger size of more than 16,641 bp, mainly due to the occurrence of multiple 84-bp tandem repeats in the IGS between *trnK* and *trnD*; however, the whole IGS was not sequenced through (see below). Like other insects, the six species showed a strong bias toward A + T, accounting for 70.34–78.77% of nucleotide composition. Moreover, highly negative GC skew values (an excess of C relative to G) and different AT skew patterns between the two subfamilies (more T for Nemobiinae and more A for Trigonidiinae) were observed (Table 1). All six mitogenomes encoded the typical set of 37 genes for insects, i.e. 13 PCGs, 22 tRNA genes, and two rRNA genes. Nine PCGs and 11 tRNA genes were encoded on the majority strand while the other genes were on the minority strand.

### Gene rearrangements and phylogenetic distribution

**Figure 1:**
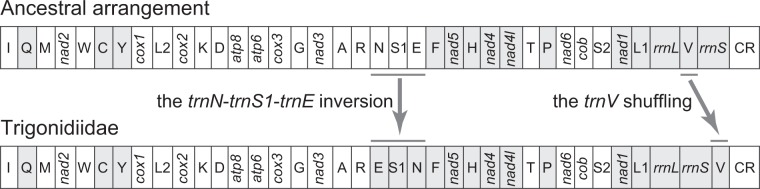
Linearized representation of mitochondrial gene order. The two types of gene rearrangements (the *trnN*-*trnS1*-*trnE* inversion and the *trnV* shuffling) in the six mitogenomes are indicated. Genes encoded on the minority strand are shaded. tRNA genes are labeled with the one-letter code for the corresponding amino acid.

**Figure 2:**
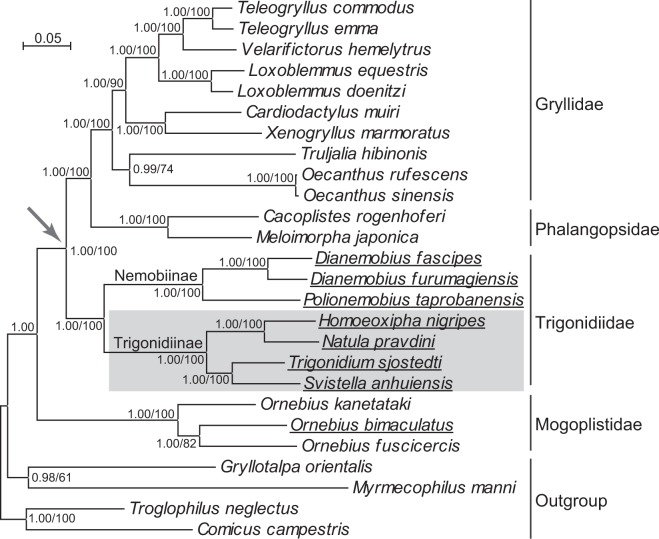
The Bayesian tree of the superfamily Grylloidea. Bayesian posterior probabilities and the maximum likelihood support values are indicated at nodes. Species with the *trnV* shuffling rearrangement are underlined, while the clade with the *trnN*-*trnS1*-*trnE* inversion is labeled with an arrow. The clade with an anticodon TCT for *trnS1* is shaded.

Relative to the ancestral gene arrangement of insects^1^, which occurs in the majority of insect mitogenomes, the six mitogenomes possessed two types of gene rearrangements including an inversion of *trnN*-*trnS1*-*trnE* encoded on the majority strand to *trnE*-*trnS1*-*trnN* on the minority strand and a shuffling of *trnV* to the position between *rrnS* and the control region (Fig. 1). The same gene rearrangements have also been observed in *T*. *sjostedti*^6^, the sole mitogenome of the family Trigonidiidae available in GenBank before our work. These species formed a monophyletic clade in the phylogenetic tree (Fig. 2), supporting the monophyly of the family Trigonidiidae^29^. The gene rearrangement pattern seems therefore to be a shared character for the family Trigonidiidae. The *trnN*-*trnS1*-*trnE* inversion has also been reported in all currently sequenced mitogenomes of two closely related families, Gryllidae and Phalangopsidae, which were supported to be sister to Trigonidiidae here (Fig. 2) and in previous studies^13,29^. Such a phylogenetic distribution pattern suggests that the *trnN*-*trnS1*-*trnE* inversion event happened in the most recent common ancestor of Gryllidae, Phalangopsidae, and Trigonidiidae, corroborating the proposal by Ma and Li^13^. Among all other sequenced mitogenomes of the suborder Ensifera, the *trnV* shuffling occurs exclusively in *O*. *bimaculatus* of the family Mogoplistidae^13^. The non-monophyly of the species with the *trnV* shuffling (Fig. 2) suggests that this rearrangement occurred independently twice in history, one in the common ancestor of Trigonidiidae and the other in *O*. *bimaculatus*.

### Protein-coding genes

Most PCGs started with ATG, ATT, and ATA, which were typical start codons for insect mitochondrial PCGs. The exceptions were *cox1* starting with CCG in all but *P*. *taprobanensis*, *nad3* with TTG in *P*. *taprobanensis*, and *nad1* with CTG in *D*. *furumagiensis* and TTG in *D*. *fascipes*, *H*. *nigripes*, *P*. *taprobanensis*, and *S*. *anhuiensis*. These anomalous start codons were proposed previously^13,30^ and among them, CCG for *cox1*^13^ and TTG for *nad1*^4,31^ have been validated by transcript information.

**Figure 3:**
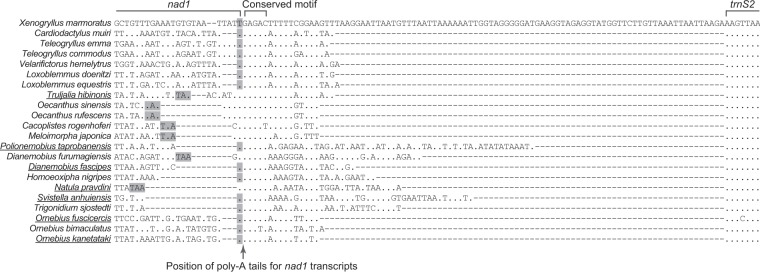
Alignments of *nad1* ends, partial *trnS2*, and the IGS. Sequences are from the minority strand. Complete (TAA and TAG) and partial stop codons (T) are highlighted in grey. The 3′ ends of *nad1* transcripts were verified by 3′ RACE and Sanger sequencing for the five underlined species in our study and two *Ornebius* species in Ma and Li^13^. The position of poly-A tails in mature *nad1* transcripts is indicated by an arrow. Dots represent nucleotides identical to those in *X*. *marmoratus*, and dashes refer to gaps in the alignment.

Both complete (TAA and TAG) and partial stop codons (T and TA) were employed by PCGs in the six mitogenomes. As in other mitogenomes, these partial stop codons always immediately preceded a tRNA gene and could be converted into a complete stop codon (TAA) by the addition of poly-A tails during polyadenylation after tRNA cleavage^4,32^. Nevertheless, it seemed problematic to annotate *nad1* stop codons based on sequence alignments alone due to high variability in their 3′-end sequences (Fig. 3) and a utilization of a partial stop codon (T) not immediately followed by a tRNA gene^13^. Since RNA data are useful to characterize the 3′-ends of mitochondrial transcripts^4,33^, 3′ RACE and Sanger sequencing of *nad1* transcripts were performed for *D*. *furumagiensis*, *N*. *pravdini*, *P*. *taprobanensis*, and *S*. *anhuiensis* in our present study. An additional species *T*. *hibinonis* was also sampled to provide further evidence. We found that they all terminated with a U plus a poly-A tail (Fig. 3), rendering the formation of a complete stop codon for *P*. *taprobanensis* and *S*. *anhuiensis*. The same observation was also noticed in the cases of two *Ornebius* species in our former study^13^. Ending at this position resulted in untranslated nucleotides in *nad1* transcripts in *D*. *furumagiensis*, *N*. *pravdini*, and *T*. *hibinonis* (Fig. 3). The presence of untranslated nucleotides is also reported in mitochondrial transcripts including *nad1* in *Drosophila*^4^. The U abutting poly-A tails corresponded to the T flanked by GAGAC, a highly conserved motif in the mitogenomes across the superfamily Grylloidea (Fig. 3). These findings suggest that the conserved motif could provide recognition signals for the precise cleavage between the U and GAGAC during mRNA maturation in Grylloidea. Although we did not find the same motif in other insects, a conserved binding site for transcription termination factor is located between *nad1* and *trnS2* in most insect orders^2,3^. It is probable that the conserved motif in Grylloidea also functions through binding the transcription termination factor to stop transcription, enabling the direct formation of the 3′-end of *nad1* mRNA. Notably, there were indeed in-frame codons TAG and TAA located between the conserved motif and downstream *trnS2* in *P*. *taprobanensis* and *S*. *anhuiensis*, respectively, but they were cleaved from mature transcripts as revealed by the 3′ RACE (Fig. 3), excluding the possibility that they acted as stop codons. Accordingly, the previously designated complete stop codons in Grylloidea, including TAA in *T*. *sjostedti*^6^, which were located downstream of the conserved motif, were expected to be removed during posttranscriptional processing. In these cases, *nad1* should be revised to terminate with the T immediately preceding the conserved motif. Collectively, we pinpoint the precise 3′-ends of *nad1* transcripts in Grylloidea and provide experimental evidence for the GAGAC-flanked T as a partial stop codon in cases of the absence of a full stop codon upstream of this motif.

**Figure 4:**
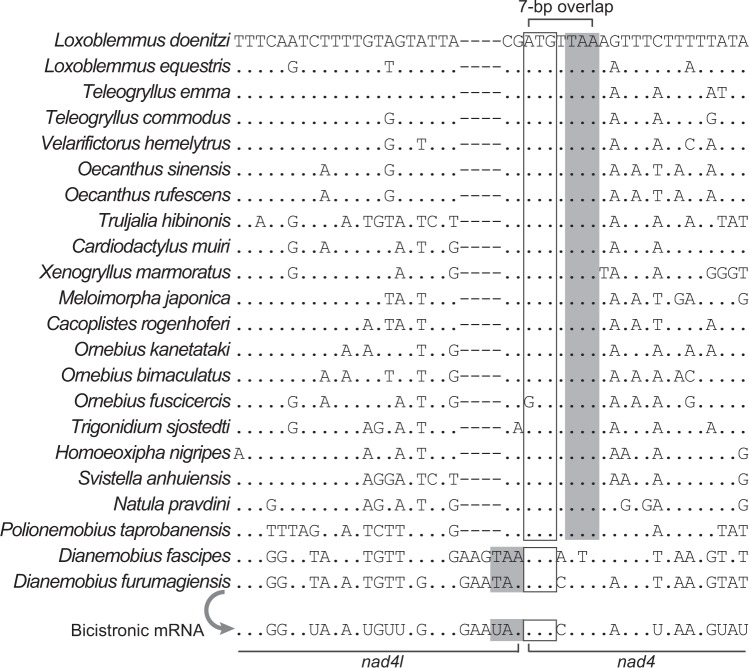
Sequence alignments of the beginning of *nad4* and the end of *nad4l*. Sequences are from the minority strand. Proposed start codons of *nad4* and stop codons of *nad4l* are boxed and shaded, respectively. In *D*. *furumagiensis*, *nad4* and *nad4l* genes exist in a bicistronic mRNA as evidenced by transcript-end sequencing.

An identical 7-bp overlap was found between *nad4* and *nad4l* in four of the six mitogenomes sequenced in our study, and the overlap was highly homologous in Grylloidea (Fig. 4). The presence of a 7-bp overlap at this location is almost a common feature in insects^3^ and the two genes can form a mature bicistronic transcript^4^. Few exceptions to the 7-bp overlap have been reported, e.g. a 40-bp IGS in a lepidopteran species^34^. Here, we found two more exceptions, i.e. no overlap or IGS between *nad4* and *nad4l* in *D*. *fascipes* and *D*. *furumagiensis*. Further, 5′ RACE of *nad4* transcript and 3′ RACE of *nad4l* transcript in *D*. *furumagiensis* supported a single mRNA comprised of the two tightly adjoining genes (Fig. 4). Our finding demonstrates that *nad4* and *nad4l* in *D*. *fascipes* and *D*. *furumagiensis* are still transcribed and processed into a mature bicistronic mRNA as in other insects^4^.

### Transfer and ribosomal RNA genes

**Figure 5:**
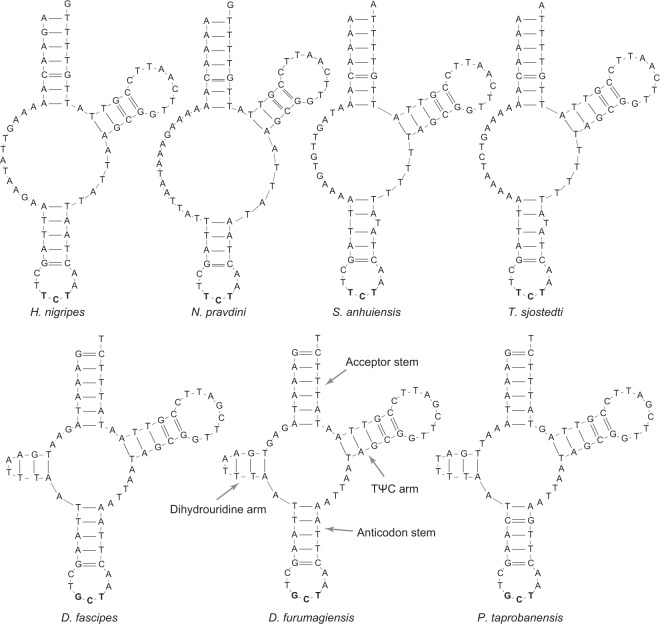
Atypical cloverleaf structures of *trnS1* in the family Trigonidiidae.

The tRNA genes ranged in size from 60 bp (*trnC* in *P*. *taprobanensis*) to 72 bp (*trnI* in *N*. *pravdini*). Compared with the regular cloverleaf secondary structure of tRNAs^35^, *trnS1* exhibited different structural features (Fig. 5). The acceptor stem was comprised of 6 bp in the three species of the subfamily Nemobiinae. The anticodon stem lacked 1 bp in *N*. *pravdini* and had an extra unpaired nucleotide in *S*. *anhuiensis* and *T*. *sjostedti*. A stable dihydrouridine arm was missing in the subfamily Trigonidiinae. Instead, it was replaced by an unpaired stretch of 15 nucleotides in *N*. *pravdini* and 12 nucleotides in *H*. *nigripes*, *S*. *anhuiensis*, and *T*. *sjostedti*. The lack of a stable dihydrouridine arm is a common feature for *trnS1* in nearly all Metazoa^36^. By contrast, the same dihydrouridine arm consisting of one G-T and two A-T pairs was observed in the three species of Nemobiinae but they all lacked a typical dihydrouridine loop.

With the exception of *trnS1*, the tRNA genes each shared the same anticodon among the six species. For *trnS1*, the anticodon was TCT in Trigonidiinae including *T*. *sjostedti*, whereas it was GCT in the three species of Nemobiinae (Fig. 5). Moreover, we found a conserved GCT anticodon for *trnS1* in all other species of the suborder Ensifera hitherto sequenced. These findings indicate that GCT is an ancestral anticodon while TCT is a derived form. Furthermore, the monophyly of Trigonidiinae in our (Fig. 2) and a previous study^29^ suggests a single G-to-T substitution event that occurred in the common ancestor of the subfamily Trigonidiinae.

The two rRNA genes (*rrnL* and *rrnS*) ranged in size from 1,273 bp to 1,312 bp and 753 bp to 797 bp, respectively. Their nucleotide composition was highly A + T-biased (73.76–79.07% for *rrnL* and 70.51–79.71% for *rrnS*). Both genes adjoined each other and were encoded by the minority strand.

### Non-coding regions

**Figure 6:**
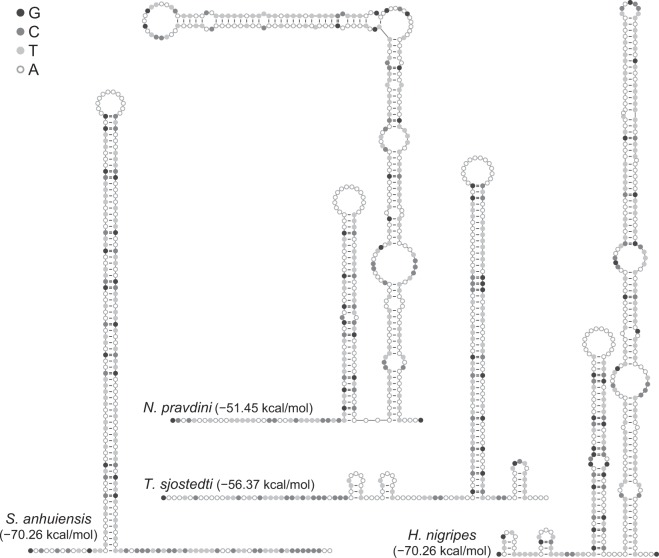
Predicted secondary structures of the IGS between *trnQ* and *trnM* in Trigonidiidae. Delta *G* values are shown in parentheses.

Non-coding regions including IGSs and the control region were identified in the six mitogenomes. Strikingly, a long IGS was detected between *trnQ* and *trnM* with a length of 210–369 bp for Trigonidiinae and 80–216 bp for Nemobiinae. Although the primary sequences varied substantially, the IGSs in Trigonidiinae were all capable of forming stable stem-loop secondary structures with a delta *G* value of −74.00~−51.45 kcal/mol (Fig. 6). A consecutive and long stem was recognized in *S*. *anhuiensis* (72 bp) and *T*. *sjostedti* (52 bp), and the stem was even longer with multiple mismatches or bulges in *H*. *nigripes* and *N*. *pravdini*. On the contrary, no obvious stem-loop structures were detected in the IGSs of Nemobiinae (delta *G* = −6.48~−1.87 kcal/mol). The IGS is 94 bp in *Xenogryllus marmoratus* (Haan, 1844)^37^ with a delta *G* of −1.67 kcal/mol and is no more than 23 bp in all other sequenced mitogenomes of Grylloidea. There is even 1-bp overlap between *trnQ* and *trnM* in *T*. *hibinonis*^38^. At a higher taxonomic level of the suborder Ensifera, the longest IGS (465 bp) between *trnQ* and *trnM* is reported in a *Deflorita* species^7^, but it could not form stable stem-loop structures (delta *G* = −15.61 kcal/mol).

The assembled control region sequences were validated by PCR amplification followed by Sanger sequencing. The control region ranged in length from 516 bp to 1,416 bp for the six species and showed a strong preference for A and T (81.12–88.45%). A 410-bp motif was found to be tandemly repeated 3.0 times in *S*. *anhuiensis*, and similar repeats larger than 100 bp were not detected in the five other species. The occurrence of tandem repeats is widely favored to be a consequence of strand slippage and mispairing during mtDNA replication^39^. The repeat motif in *S*. *anhuiensis* was capable of forming multiple stem-loop secondary structures (delta *G* = −42.61 kcal/mol), which could enhance the probability of replication slippage by stabilizing slipped strands or blocking the polymerase^40^.

A novel non-coding region larger than 893 bp was exclusively identified between *trnK* and *trnD* in *P*. *taprobanensis*. More than five 84-bp tandem repeats were sequenced at each end of this region but the entire repeat region was not sequenced through with short-read data from Illumina sequencing. It is suggested to validate non-coding regions possibly containing repeats via PCR amplification followed by Sanger sequencing^13^. After many attempts, however, we failed to amplify this region. Instead, we obtained a massive smear of electrophoretic bands, a prevalent observation for PCR amplification of repetitive DNAs^41^. The exact copy number of these repeats remained therefore unknown.

## Conclusion

Comparative analysis of the six representative mitogenomes of the family Trigonidiidae and those from related species reveals common and unique structural features including gene rearrangements, large-sized IGSs with secondary structures, altered tRNA anticodons, and no overlap between adjacent *nad4* and *nad4l*. These findings add to our knowledge of structural diversity of insect mitogenomes. Transcript end sequencing demonstrates that mature *nad1* mRNA terminates with a U, corresponding to the T immediately followed by a highly conserved motif GAGAC in Grylloidea, plus a poly-A tail generated through polyadenylation. The GAGAC-flanked T acts as a partial stop codon in cases where a full stop codon upstream of this motif lacks. Our study thus provides a guide toward reliable annotation of *nad1* stop codons in Grylloidea and has implications for understanding the process of mitochondrial transcript maturation.

## Supplementary information

Table S1

## Data Availability

The datasets generated during the current study are available from the corresponding author on reasonable request.
