# Supplementary material for: Mitochondrial genome characterization of the family Trigonidiidae (Orthoptera) reveals novel structural features and nad1 transcript ends
Source: Sci Rep. 2019 Dec 13;9:19092. doi: 10.1038/s41598-019-55740-4 (PMC6911046; doi:10.1038/s41598-019-55740-4)
Supplement: Supplementary file 1 — Table S1 [file 41598_2019_55740_MOESM1_ESM.pdf]

Mitochondrial genome characterization of the family Trigonidiidae (Orthoptera) reveals novel structural features and *nad1* transcript ends

Chuan Ma[1,#], Yeying Wang[2,#], Licui Zhang[1], Jianke Li[1,*].

[1]*Institute of Apicultural Research/Key Laboratory of Pollinating Insect Biology, Ministry of Agriculture, Chinese Academy of Agricultural Sciences, Beijing 100093, China.*

[2]*Key Laboratory of State Forestry Administration on Biodiversity Conservation in Karst Mountainous Areas of Southwestern China, Guizhou Normal University, Guiyang 550025, China*

[#] These authors contributed equally to this work.

[*]Corresponding Author: Jianke Li (apislijk@126.com)

Table S1. PCR primer sequences used to amplify the control region and *nad1* transcript ends.

Species	Primer name	Sequence (5'-3')
Homoeoxipha nigripes	14233f	CATCTACTTTGTTACGACTT
	602r	GTGAGAACTGAACCGAGG
Natula pravdini	14635f	AAGGATAAAATACCGTCAAA
	797r	TGATGAGGATATGGCTTG
	12512r-nad1-3'RACE	GGGAAGGTTACGTTCTGTTGCTC
Svistella anhuiensis	14532f	GTACTATCAATTACAGCACA
	407r	TTCTATTTTCGGGGTATGA
	12187r-nad1-3'RACE	ATGTTGAGTATAGAAGAGGAGGATT
Dianemobius fascipes	14186f	GCACCTTGACCTGACATAC
	385r	TCACATAGCGACTCACGAA
Dianemobius furumagiensis	14205f	GCACCTTGACCTGACATA
	468r	TTAACGAGGCTTCAGACG
	12201r-nad1-3'RACE	CTGTCGCACAAACGATTTCTTATG
	9116f-nad4-5'RACE	TGAATACGCTCAGGCTGATACCC
	9780r-nad4l-3'RACE	AGAGGAATATGGGTATTTTGTAGAA
Polionemobius taprobanensis	11048f	CGGACTAAAACACCGCCAA
	13383r	GTAAGAATCCAATGAAAGGG
	8793r-nad1-3'RACE	TTGTTGGTTTTCTTCTTGTTTAGCG
Truljalia hibinonis	12018r-nad1-3'RACE	GATCTGTGGCACAAACTATTTCTTAT
